# How to build a grid cell

**DOI:** 10.1098/rstb.2012.0520

**Published:** 2014-02-05

**Authors:** Christoph Schmidt-Hieber, Michael Häusser

**Affiliations:** Wolfson Institute for Biomedical Research and Department of Neuroscience, Physiology and Pharmacology, University College London, Gower Street, London WC1E 6BT, UK

**Keywords:** grid cell, entorhinal cortex, spatial navigation, patch clamp, neural circuit, path integration

## Abstract

Neurons in the medial entorhinal cortex fire action potentials at regular spatial intervals, creating a striking grid-like pattern of spike rates spanning the whole environment of a navigating animal. This remarkable spatial code may represent a neural map for path integration. Recent advances using patch-clamp recordings from entorhinal cortex neurons *in vitro* and *in vivo* have revealed how the microcircuitry in the medial entorhinal cortex may contribute to grid cell firing patterns, and how grid cells may transform synaptic inputs into spike output during firing field crossings. These new findings provide key insights into the ingredients necessary to build a grid cell.

## 1. Introduction

An accurate representation of space is critical for an animal's survival. How does the brain accomplish this task at the level of single neurons and neuronal circuits? The mammalian hippocampus and entorhinal cortex contain neurons exhibiting spatially selective action potential firing: place cells, which are mainly found in hippocampal areas CA1 and CA3, typically fire at a single spatial location in an environment [1]. In contrast, grid cells exhibit multiple firing locations that form the vertices of a periodic triangular array covering the entire spatial environment of an animal [2]. Most principal neurons in layer II of the medial entorhinal cortex (MEC II) show pure grid firing lacking other types of spatial modulation, such as head-direction sensitivity [3], and grid-like firing behaviour can be found in both types of principal neurons in MEC II, stellate and pyramidal cells [4]. Grid cells have most extensively been studied in rats and mice, but more recent evidence has also revealed grid cells in bats [5] and primates [6] including humans [7]. In this review, we focus on the mechanisms that generate this striking grid cell code, which could be used by mammals to estimate their spatial location from self-motion information, without requiring external cues, in a process termed ‘path integration’ [2,8,9].

As an animal approaches the centre of a grid cell firing field, spikes increase in frequency, thereby encoding spatial position by spike rate. In addition to this rate code, spikes in MEC II grid cells occur at successively earlier phases of extracellular oscillations in the theta frequency band (5–12 Hz) during a grid field traversal [10], giving rise to an independent temporal code in which the amount of phase precession conveys a more precise measure of animal position than the spike rate code [11]. The resilience of phase precession in grid cells to hippocampal inactivation [10] suggests that the MEC might drive phase precession in the hippocampus, where it was first described 20 years ago [12].

Various models have been proposed to explain how the striking grid cell firing pattern arises from network connectivity, synaptic mechanisms and intrinsic membrane properties. These models have been broadly classified into oscillatory models and network models in the past; however, a more detailed and accurate classification has recently been proposed that dissects models according to how positional information is encoded, updated and read out [13].

Oscillatory interference models were originally developed to explain the rate and temporal code of hippocampal place cells [12]. In oscillatory interference models of place cells, a somatic conductance that oscillates at the theta frequency of the local field potential (LFP) interacts with a faster dendritic oscillation that depends linearly on animal speed [12,14], resulting in a compound somatic membrane potential oscillation (MPO). Spikes occur whenever this compound MPO exceeds action potential threshold and will therefore show phase precession because the compound MPO frequency is greater than extracellular theta. The amplitude envelope of the compound MPO, which is modulated by a slow beat frequency, defines place field locations and dimensions.

To account for grid cell firing in a two-dimensional environment, a revised oscillatory interference model was proposed [15–17]. In the grid cell model, rather than employing a single dendritic speed-controlled oscillator, two or more dendritic velocity-controlled oscillators (VCOs) are differentially controlled by animal speed and running direction. Early model implementations suggested that the dendritic VCOs might originate from intrinsic dendritic membrane properties [16]. However, modelling showed that dendrites are unlikely to oscillate independently at different frequencies over sufficiently long periods of time [18], making it more likely that the VCOs are driven by synaptic inputs from direction-sensitive neurons [15,19]. At any rate, action potential firing in this type of model is produced when the VCOs are in phase with each other. Thus, the fundamental firing mechanism consists of rapid coincidence detection of VCO phases within theta cycles [15,20].

Continuous attractor network (CAN) models make up the majority of network-level models that have been developed to explain grid cell firing. They were originally proposed for place and head-direction cells [21,22] and have later been extended to grid cells [8,9,23]. In these models, neurons are arranged on a neural sheet according to where they fire in a two-dimensional environment. The strength of symmetric recurrent synaptic connections declines with distance between neurons in either a graded [9,23] or an all-or-none [24] fashion. Such a centre-surround synaptic weight profile provides the neural sheet with continuous attractor properties and can lead to spontaneous formation of a periodic array of activity clusters. To couple animal movement to neural activity, an additional hidden layer of velocity-sensitive neurons is asymmetrically connected to the continuous attractor layer so that the activity clusters are shifted as a function of animal movement. Firing in this model is produced when a neuron becomes part of an activity cluster, and the fundamental firing mechanism consists of a sustained net increase in excitation during a firing field crossing.

The various proposed models highlight that there exist multiple plausible mechanisms for generating a grid-like firing pattern. Which mechanisms are actually used in the real entorhinal circuit? Over the past year, a series of complementary studies have probed the mechanistic basis of grid cell firing in unprecedented detail. In particular, *in vitro* experiments have provided new data about the connectivity of the MEC circuit [24,25] and *in vivo* whole-cell recordings in navigating animals [4,26] have offered the first glimpse into the transformations of synaptic input to spike output that give rise to grid cell firing. Together, these studies have yielded crucial information about the cellular, synaptic and circuit ‘toolkit’ that the medial entorhinal cortex uses to build grid cells.

## 2. Intrinsic membrane properties

Stellate cells are the main principal neuron type in MEC II [27]: approximately 70% of the total MEC II neuron population are stellate cells, whereas pyramidal neurons make up only approximately 15% [28]. Up to 50% of extracellularly recorded neurons in MEC II display pure grid cell firing patterns without head-direction tuning [3], and stellate cells have recently been shown to exhibit grid-like firing patterns in mice navigating on a virtual linear track [4,26]. Moreover, a recent study has used an optogenetic strategy to show that grid cells in MEC layer II can project to hippocampus [29], as do layer II stellate cells [30,31]. It is therefore likely that stellate cells represent a large fraction of the grid cell population, but given that neurons in other layers also show grid cell firing, it is clear that the grid cell phenotype is not restricted to a single cell type. Stellate cells have received substantial attention even before the discovery of grid cells because of their distinctive intrinsic excitability and responsiveness in the theta frequency range. The remarkable intrinsic membrane properties of these neurons are therefore of particular interest when linking cellular mechanisms to grid cell firing [32].

Stellate cells express a high density of hyperpolarization-activated cyclic-nucleotide-gated (HCN) channels, which dominate the resting conductance [33]. The resulting low input resistance of stellate cells both *in vitro* [32,34] and *in vivo* [26,35] places an important constraint on the minimum number of inputs required for generating output spikes during grid cell firing. The high level of HCN channel expression also has important implications for dynamic membrane properties. When sinusoidal current waveforms of varying frequencies are injected into rodent stellate cells *in vitro*, the membrane potential response shows pronounced resonance in the theta frequency range [36–38], and spikes are phase-locked to theta inputs under a variety of synaptic input scenarios [39]. This phase-locking of spikes to theta is critically dependent on HCN channels [39]. By contrast, non-stellate cells in rodent MEC II/III lack subthreshold theta frequency resonance [36–38]. Intriguingly, stellate cells in bats also exhibit no such resonance [40], accompanying a lack of continuous extracellular theta oscillations during grid cell firing in this species [5]. Stellate cells in rodents can also spontaneously produce pronounced intrinsic MPOs in the theta frequency range when depolarized close to spike threshold by steady-state current injections *in vitro* [27,41]. It has been suggested that these oscillations may be caused by the interplay between persistent sodium channels and HCN channels [42,43]. Alternatively, more recent work suggests that they can be explained by stochastic gating of voltage-gated channels [37,44–47].

The frequency of these intrinsic MPOs depends on membrane potential and on the dorsal–ventral location of a stellate cell within MEC II [48], paralleling a gradient that has been found in grid field spacing along the same dorsal–ventral axis [2]. Because of the similarity of these gradients, intrinsic MPOs were incorporated into some oscillatory interference models of grid cell firing [48]. However, recent experimental and modelling studies have noted that the frequency of intrinsic MPOs is neither sufficiently tuned nor sufficiently stable over longer periods of time to support robust oscillatory interference [32,44,49]. Moreover, subthreshold depolarization of stellate cells in awake resting animals fails to evoke significant theta MPOs [26]. This is consistent with the observation that strong spontaneous synaptic input dampens these oscillations *in vitro* [50], making it unlikely that intrinsic MPOs are directly involved in grid cell firing.

The gradient in intrinsic MPO frequencies reflects a gradient in intrinsic membrane properties that determines the temporal integration properties of stellate cells. Dorsal–ventral differences in the HCN channel density and leak potassium conductance generate a dorsal–ventral gradient in a number of intrinsic membrane properties of stellate cells, such as input resistance, membrane time constant and membrane potential sag [34,48,51]. Moreover, as a consequence of the gradient of intrinsic membrane properties, dorsal cells exhibit a shorter time window for synaptic integration and less temporal summation of excitatory postsynaptic potentials in the gamma-frequency range than ventral cells, leading to the suggestion that synaptic integrative properties of stellate cells are tuned to the complementary dorsal–ventral gradient of grid field spacing [34]. In good agreement with this hypothesis, knockout of the HCN1 subunit causes an expansion of grid field spacing and size, suggesting that these channels are involved in setting the gain of velocity signals to grid cells [52]. This study is particularly important as it is the first to directly assess the effects of intrinsic membrane properties on grid cell firing.

## 3. Network connectivity

**Figure 1.**
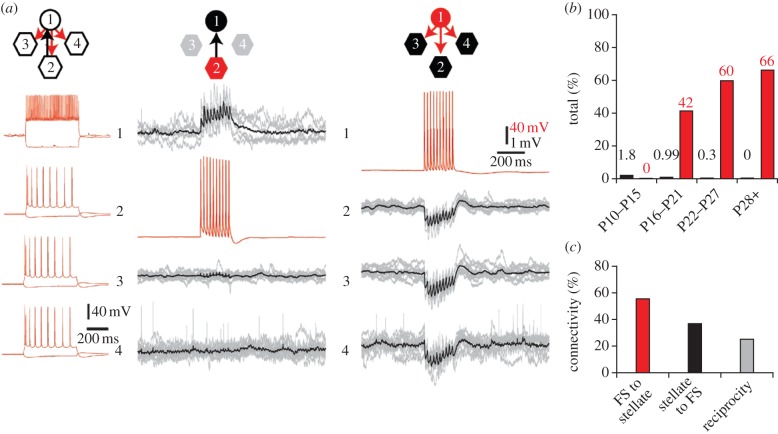
Recurrent connectivity in MEC II is provided by interneurons. (*a*) Quadruple whole-cell recording *in vitro* from a fast-spiking interneuron (1) and three stellate cells (2, 3 and 4). Only the interneuron responds with excitatory postsynaptic potentials to the stimulation of a stellate cell (middle). By contrast, all stellate cells display inhibitory responses when the interneuron is stimulated (right). (*b*) Connectivity rates for excitation (black) and recurrent inhibition (red) as a function of postnatal age. Note near-absence of excitatory connections in adult animals. (*c*) Connectivity rates for inhibitory connections from fast-spiking to stellate cells, excitatory connections from stellate to fast-spiking cells, and the reciprocity of these connections. ((*a*–*c*) Adapted with permission from Couey *et al*. [24].)

Recent studies using simultaneous intracellular recordings from multiple stellate neurons and targeted optogenetic activation of MEC neurons *in vitro* have greatly contributed to our understanding of the functional connectivity within the MEC microcircuit [24,25] (figure 1). Surprisingly, and in contrast to other cortical regions, recurrent excitatory connections appear to be rare in MEC, a finding that is supported by multiple lines of evidence. First, optogenetic stimulation of MEC II principal cells [24] or stellate cells and interneurons [25] results almost exclusively in inhibitory responses in stellate cells. Similarly, simultaneous intracellular recordings from multiple stellate cells fail to detect any direct excitatory connections, while stimulation of interneurons reliably evokes monosynaptic inhibitory postsynaptic responses in stellate cells [24] (figure 1*a*). Therefore, these studies indicate that stellate cells are mainly interconnected by inhibitory interneurons, while recurrent excitation is sparse or absent (figure 1*b*,*c*). This makes a striking contrast to other cortical areas in the mammalian brain, where recurrent excitatory feedback is prominent [53]. Feedback inhibition may also serve as a clock signal for the temporal grid cell code, as optical stimulation of MEC II at theta frequency produces nested gamma-frequency synaptic activation in stellate cells [25]. By contrast to stellate cells, MEC layer II pyramidal cells, some of which have also been shown to produce grid-like firing [4], receive direct excitatory inputs when MEC layer II principal neurons are optically stimulated [24]. This raises the interesting possibility that stellate cells may entrain pyramidal cells to produce grid firing.

**Figure 2.**
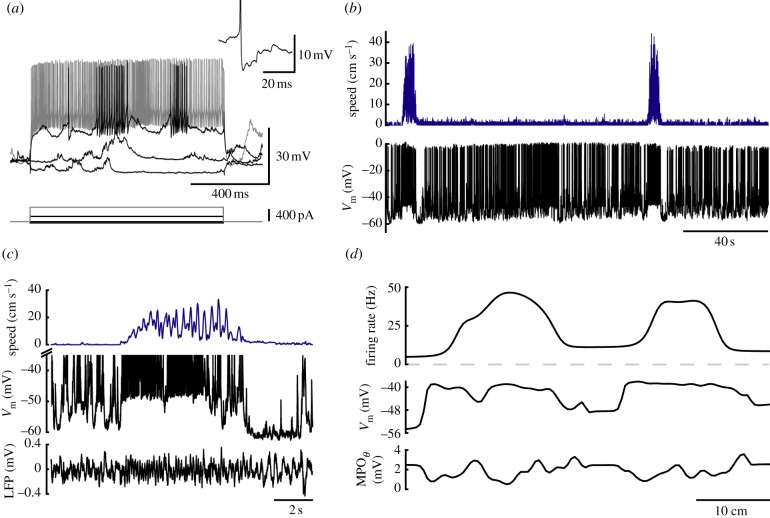
Membrane potential dynamics of fast-spiking neurons in the MEC of navigating mice. (*a*) Electrophysiological characterization of a fast-spiking neuron from MEC of an awake mouse. The inset shows a representative action potential. (*b*) Animal speed (top) and membrane potential (bottom) of the fast-spiking cell shown in (*a*). (*c*) Animal speed (top), membrane potential (middle) and LFP (bottom) during the first movement period in (*b*) plotted at higher magnification. (*d*) Firing rate (top), subthreshold membrane potential (middle) and theta MPO amplitude were plotted against the position of the animal along the long axis of the track. High basal firing rates and spatial modulation of firing are consistent with the predictions for inhibitory conductances by CAN models. ((*a*–*c*) Adapted from Schmidt-Hieber & Häusser [26].)

A direct consequence of some CAN models that build on purely inhibitory recurrent connectivity [24] is the prediction that some MEC interneurons should exhibit grid-like spatial firing patterns, because grid cells with similar phases must ‘share’ an inhibitory interneuron. However, while these recent studies have provided important new data on the functional connectivity between interneurons and principal neurons in MEC II, little is known about spatial modulation of firing in MEC interneurons, partly because it is difficult to reliably identify interneurons from the spike waveform in extracellular recordings. While whole-cell recordings from fast-spiking neurons in MEC indicate that interneuron firing may be spatially modulated [26] (figure 2), recordings from larger populations of identified interneurons in navigating animals will be required to fully establish how interneurons contribute to grid cell firing.

## 4. Synaptic inputs

**Figure 3.**
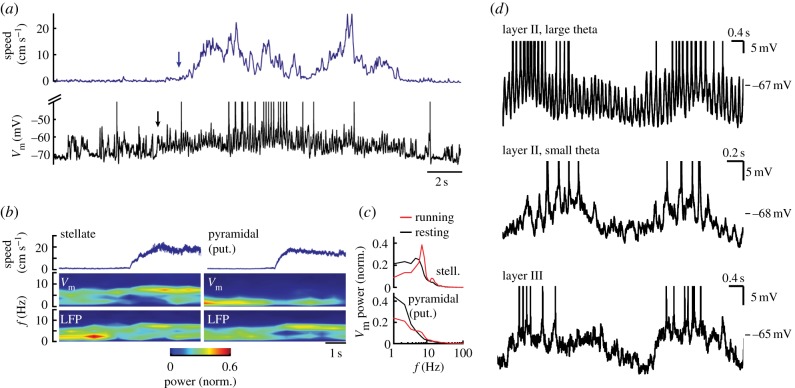
Membrane potential of stellate cells shows theta periodicity during running. Whole-cell recordings from MEC II of mice navigating on a spherical treadmill. (*a*) Mouse speed (blue) and membrane potential of a stellate cell (black) during a running period. Note that onset of oscillatory activity (black arrow) precedes onset of running (blue arrow). (*b*) Spectrograms of membrane potential (middle) and LFP (bottom) were aligned to the onset of movement (top) before computing the average for stellate cells (left) and putative pyramidal cells (right). (*c*) Average power spectra for stellate cells (top) and putative pyramidal cells (bottom) at rest (black) and while running (red). Note the distinct theta peak in the power spectrum of stellate cells during running. ((*a*–*c*) Adapted from Schmidt-Hieber & Häusser [26].) (*d*) Membrane potential traces of an MEC II neuron with large theta oscillations (top), an MEC II neuron with small theta oscillations (middle) and an MEC III neuron (bottom). Theta oscillation amplitudes range from 2 to 12 mV. (Adapted with permission from Domnisoru *et al*. [4].)

What type of synaptic inputs do grid cells receive *in vivo*? Addressing this question requires intracellular recordings from MEC neurons *in vivo* in anaesthetized and awake animals, which has only recently become possible [4,26,35,54]. Stellate cells show strong theta periodicity of membrane potential during LFP theta periods *in vivo* in anaesthetized rats [35] and during running periods in navigating animals [4,26] (figure 3), while this type of theta activity is mostly absent in non-stellate neurons from deeper layers of MEC [4,26,35]. In addition, pyramidal neurons in layer II of MEC have also been found to show pronounced theta periodicity [4]. Given that sustained depolarization of stellate cells fails to evoke theta MPOs *in vivo* in resting mice [26], theta periodicity of membrane potential during running periods is likely generated by theta-modulated synaptic activity, and may be enhanced or facilitated by neuromodulatory inputs. The absence of substantial theta MPOs during running in non-stellate neurons from deeper MEC layers is intriguing, as it is paralleled by a lack of robust phase precession in extracellularly recorded MEC III grid cells [10]. It might be explained by selective targeting of MEC II by theta-modulated inputs, or alternatively, theta resonance in MEC II principal neurons may selectively amplify these inputs.

What is the source for theta input to MEC? Several lines of evidence point to the medial septum and diagonal band of Broca as possible candidates. Neurons in these regions, from which MEC is known to receive inputs [55], fire theta-modulated bursts during locomotion [56]. Moreover, inactivating the medial septum abolishes theta rhythmic firing in MEC [57] and makes grid cells lose their spatial periodicity [58,59]. Theta in the MEC is largely resistant to cholinergic blockade [57], and cholinergic stimulation decreases theta responsiveness of stellate cells *in vitro* [60,61], making it unlikely that theta in the MEC is generated by cholinergic projections from the medial septum. In the hippocampus, theta rhythmic drive is thought to be provided by GABAergic inputs from parvalbumin-expressing neurons in the medial septum [62,63], which are known to target hippocampal inhibitory interneurons [64], causing disinhibition of pyramidal cells [65]. Whether a similar circuit for theta pacemaking exists between the medial septum and the MEC is unclear. Further studies will be required to answer this question.

**Figure 4.**
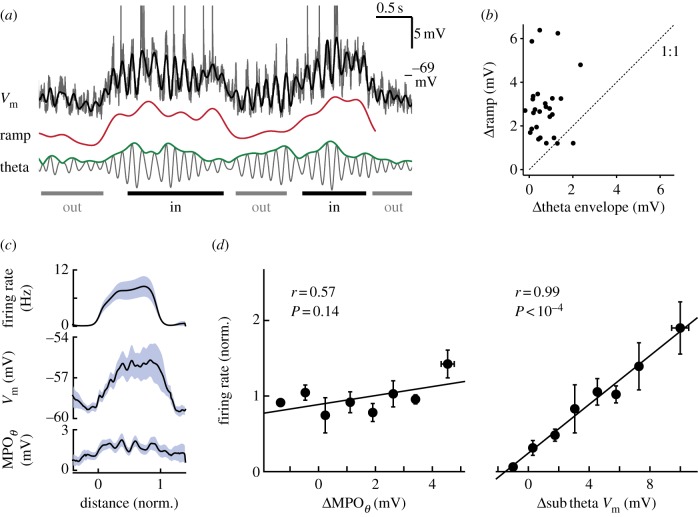
Grid cell firing is driven by sustained depolarizations. Whole-cell recordings from MEC of mice navigating on a spherical treadmill. (*a*) Membrane potential of an MEC neuron (black) during a run along a linear track. Two firing fields were crossed, as indicated at the bottom. Membrane potential was decomposed into a ramp (red) and a theta oscillation (grey). Note the sustained increase in membrane potential during field crossings. (*b*) The ramp voltage increased more than theta oscillation amplitude during firing field crossings, indicating that sustained depolarizations drive grid cell firing. ((*a*,*b*) Adapted with permission from Domnisoru *et al*. [4].) (*c*) Average firing rate (top), subthreshold membrane potential (middle) and theta MPO amplitude (bottom) were plotted against normalized position in a firing field of a stellate cell. Theta MPOs contributed only little to the depolarization in the field centre. (*d*) Normalized firing rates of stellate cells were plotted against deviations of theta MPO amplitudes (left) and subtheta membrane potential (right) from the mean. By contrast to subtheta membrane potential changes, changes in theta MPO amplitudes did not significantly correlate with firing rates, indicating that firing was primarily driven by slow depolarization. ((*c*,*d*) Adapted from Schmidt-Hieber & Häusser [26].)

When animals cross the firing field of a stellate cell, membrane potential shows a sustained depolarization driving spike output [4,26] (figure 4), similar to what has been described for place cells in hippocampal area CA1 [66]. Non-stellate cells with grid-like firing also display this slow depolarization [4], suggesting a conserved firing mechanism across all cell types that can produce grid firing. This slow depolarization determines the spike rate during a field crossing, while theta MPOs impose spike timing. Given the dense recurrent inhibitory connectivity that has recently been described, it seems plausible to speculate that this depolarization is at least in part caused by a reduction in inhibition. Excitatory inputs from deeper layers of MEC, potentially driven by hippocampal projections [67], may then dominate during firing field crossings. Voltage-clamp experiments in behaving animals will be required to test this hypothesis [68].

Sensory inputs are known to affect grid cell firing. Environmental novelty, for example, causes the grid scale to expand, which might constitute a mechanism for updating hippocampal representations [69]. Sensory inputs might also reset the path integration mechanism to prevent error accumulation [15]. The source of these inputs has not been experimentally determined yet, but it has been suggested that information about distance to boundaries might reach the MEC via hippocampal place cells [70]. Regardless of their source, it seems likely that sensory inputs contribute to the observed ramp depolarization.

It is unclear how the synaptic inputs underlying the shift in excitation–inhibition balance are spatially and temporally integrated in the dendritic tree of grid cells. It is now well established that neurons are not just simple devices that linearly sum up synaptic inputs, as is largely assumed in network-level implementations of grid cell firing models. Active voltage-dependent conductances confer strongly nonlinear computational capabilities to the dendritic tree of a neuron [71,72]. While such nonlinearities have fundamental implications for how any model of grid cell firing could be implemented, only very little is known about active spatial and temporal dendritic integration in stellate cells [73]. For example, models of phase precession will critically depend on how active conductances contribute to synaptic integration time windows within theta periods, in particular if the model builds on separate oscillatory processes in the dendritic and somatic domains [18,74–76]. Therefore, it will be essential to assess how synaptic inputs are spatially and temporally integrated throughout the dendritic tree of grid cells.

## 5. Constraints for grid cell models

### (a) Rate code

The slow depolarization driving grid cell firing that has recently been observed *in vivo* argues in favour of slow shifts of excitation–inhibition balance generating the rate code of grid cell firing (figure 4). Such a slow shift is inconsistent with models that rely on rapid coincidence detection of synaptic inputs during theta cycles, for example the oscillatory interference model. Stellate cells have a fast membrane time constant (less than 20 ms *in vitro* [34]), and spikes are followed by afterhyperpolarizations [43], leading to rapid and reliable resetting of membrane potential within less than a theta period. As a consequence, it is difficult to produce the net, slow depolarization observed experimentally using single-cell oscillatory interference models [26].

Among the models of grid cell firing that have been proposed, a CAN model predicts a slow depolarization during firing that is consistent with the experimental data. Several recent lines of evidence provide additional support for this model. The strong reciprocal connections between interneurons and stellate cells can provide the recurrent connectivity that is required for CAN models [24,25]. Notably, even unstructured all-or-none recurrent inhibitory connectivity, as predicted by the experimental data, is sufficient to generate grid-like firing patterns in CAN models [24]. Feed-forward excitation may be provided by hippocampal inputs, which have been shown to be required for grid cell periodicity [67], either indirectly via neurons from deeper layers of MEC that receive hippocampal projections [77,78] or directly by CA2 projections to superficial MEC layers [79]. The observation that grid spacing increases in discrete steps rather than continuously along the dorsal–ventral axis could indicate that grid cells are organized into modular continuous attractor subnetworks within MEC [80–82]. Such a modular organization of the network also argues against single-cell models that solely rely on intracellular mechanisms to generate grid cell firing.

### (b) Temporal code

**Figure 5.**
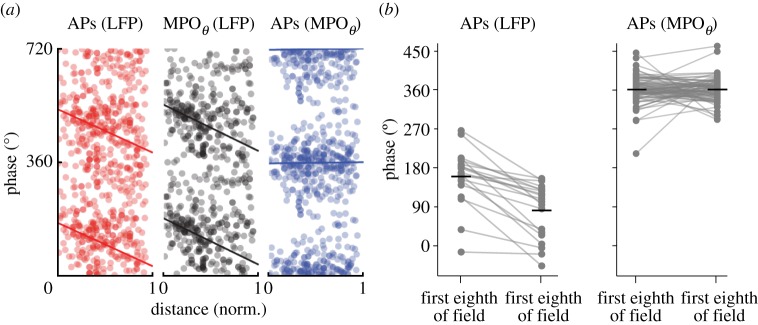
Action potentials are in phase with MPOs during grid field crossings. (*a*) The phases of action potentials (APs) with respect to LFP theta (left), theta MPOs with respect to LFP theta (middle) and action potentials with respect to MPOs (right) were plotted as a function of normalized position within firing fields of stellate cells. (Adapted from Schmidt-Hieber & Häusser [26].) (*b*) Mean phase of APs with respect to LFP theta (left) and with respect to theta MPOs (right) in the first and last eighth of each field. Action potentials showed no significant phase precession with respect to theta MPOs. (Adapted with permission from Domnisoru *et al*. [4].)

**Figure 6.**
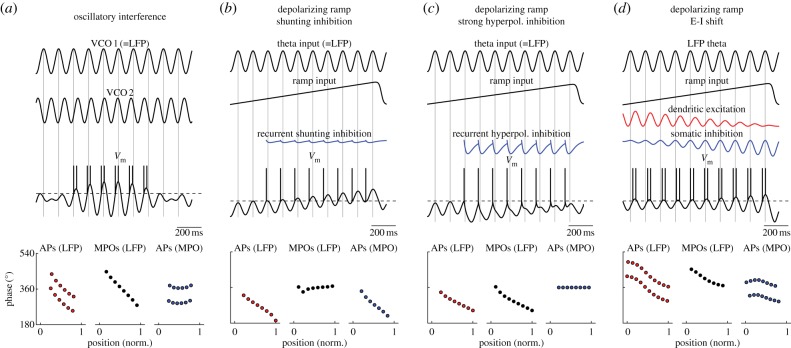
Comparing models of phase precession to experimental data. (*a*–*d*) Schematic drawings of phase precession models. Phase precession (bottom) is plotted as in figure 5*a*. (*a*) An oscillatory interference model with two VCO inputs correctly predicts the experimentally observed phase precession. (*b*) A depolarizing ramp model with theta input phase-locked to LFP theta produces phase precession of APs with respect to LFP theta. Shunting recurrent inhibition prevents repetitive firing within theta periods, and thereby sharpens phase precession. However, APs show phase precession with respect to LFP theta, contrary to the experimental data. (*c*) Strongly hyperpolarizing recurrent inhibition abbreviates theta MPOs so that the experimental phase precession is reproduced. (*d*) Both dendritic excitatory inputs and somatic inhibitory inputs are phase-locked to LFP theta, but phase-shifted by 60° against each other. During the depolarizing ramp, excitatory drive decreases and inhibitory drive increases because of opposing changes in driving force. This shift in excitation–inhibition balance can produce phase precession of action potentials with respect to LFP theta [83].

Both spikes and theta MPOs show the same amount of phase precession with respect to LFP theta in grid cells; as a consequence, spikes and theta MPOs are in phase when an animal crosses a firing field [4,26] (figure 5). Oscillatory interference (OI) models can produce phase precession if the VCOs are directionally tuned so that their firing frequency is always greater than LFP theta [15]. In this case, OI models can reproduce all aspects of phase precession, including phase precession of MPOs [20,26] (figure 6*a*). By contrast, models of phase precession using synaptic inputs that are phase-locked with LFP theta are more difficult to reconcile with phase precession of both MPOs and spikes with respect to LFP theta [20]. As an example, a depolarizing ramp model that has originally been proposed for phase precession in place cells combines a ramp of excitatory drive with synaptic inputs that are modulated at LFP theta frequency [84] (figure 6*b–d*). The depolarizing ramp makes theta MPOs cross spike threshold increasingly earlier during each theta cycle, causing phase precession of spiking with respect to LFP theta. Recurrent inhibition prevents repetitive firing during theta cycles, and thereby sharpens phase precession in this model. When implemented in a compartmental model of a stellate cell using predominantly shunting recurrent inhibition, this model predicts that MPOs will be in phase with LFP theta [26], contrary to the experimental results (figure 6*b*). However, depolarizing ramp models using strongly hyperpolarizing recurrent inhibition (figure 6*c*) or combining theta-modulated excitatory and inhibitory inputs [83] (figure 6*d*) may better fit the experimental data. Other phase precession models using synaptic inputs that are phase-locked to LFP theta, such as somato-dendritic interference models [74–76] and models that combine theta inputs, network connectivity and action potential dynamics to produce phase precession [85], remain to be tested in detail for their compatibility with phase precession of theta MPOs.

Given that no single model is currently able to simultaneously explain both the rate and the temporal code of grid cell firing, one may speculate that different mechanisms account for these phenomena, and several models that combine theta inputs and attractor dynamics have recently been proposed [26,85–88]. As oscillatory interference is a mechanism to read out spatial position, while a CAN serves the different purpose of encoding and maintaining this positional information [13], it is not surprising that these models are not mutually exclusive. Accordingly, early implementations of oscillatory interference models already suggested that neurons receiving inputs from directionally tuned VCOs could be part of a CAN with recurrent symmetric connectivity [15], which could capture both the rate and the temporal grid cell code [26]. In such a hybrid model, the path integration mechanism that couples the neural activity pattern to animal movement could be provided by asymmetric inputs from an additional layer of velocity-sensitive neurons or by the phase relationships of theta-modulated VCOs. However, the observation that some species lack continuous extracellular theta oscillations and theta spike modulation during place and grid cell firing [5,6,89] favours a mechanism that is independent of oscillatory processes in the theta frequency band but does not rule out that oscillatory interference may occur at lower frequencies [90].

## 6. Conclusion

Grid cell firing can be produced by a wide range of plausible mechanisms that can be captured by computational models. However, grid cell firing in the brain is constrained by the physical properties of the neurons involved and by their patterns of connectivity. In this review, we have focused on the cellular, synaptic and network ingredients used to generate grid cell firing in the mammalian entorhinal cortex. At each level, these ingredients are surprisingly specialized, which limits the potential range of mechanisms available, and thus provides crucial constraints for models of grid cell function. Moreover, these cellular and network ‘building blocks’ can help us to identify (or rule out) potential grid cells in other brain areas.

On the cellular level, stellate cells, which likely represent a large fraction of the grid cell population, show highly specialized intrinsic membrane properties, including low input resistance, pronounced responsiveness in the theta frequency range and rapid membrane time constants, which are tuned to their grid cell function. While we have focused on stellate cells in this review, it will also be interesting to determine which ingredients of intrinsic and synaptic properties drive spatially modulated firing in non-stellate grid cells in the entorhinal cortex. On the level of network connectivity, the MEC exhibits a distinctive wiring diagram, lacking prominent recurrent excitation between stellate cells and being dominated by strong recurrent inhibition. Finally, although the spatio-temporal dynamics of MEC population activity remain almost entirely unexplored, it is clear that a complex interaction between theta-modulated inputs, velocity-dependent inputs and a source of sustained excitation are required to generate the observed grid cell pattern.

Knowing these properties of the mammalian MEC allows us to either rule out certain classes of grid cell models, or to make biophysically realistic predictions, for example for intracellular membrane potential trajectories, that can be used to discriminate between different models. Intracellular recordings from grid cells in navigating animals, which directly test these predictions, show that the intracellular signature of grid cell firing is a sustained increase in net excitation—consistent with CAN models—while theta MPOs define the temporal structure of the grid cell code.

However, demonstrating consistency with CAN models is insufficient for proving that such models provide an accurate and unique description of grid cell firing in the MEC. For example, sustained depolarizations during grid field crossings are not necessarily a unique prediction of CAN models. Other network models may fit the data equally well, but have not yet been implemented in a way that would allow one to make realistic predictions of membrane potential dynamics [13,91]. Simultaneous recordings from pairs of grid cells over time and across environmental manipulations are beginning to provide more specific evidence for CAN dynamics [92]. However, future experiments recording from large populations of identified principal and interneurons in the entorhinal cortex of behaving animals [93] are required to identify which neurons are spatially modulated and whether the spatio-temporal dynamics of the interactions between the various identified cell types fully conforms to predictions made by the CAN models. Ultimately, targeted perturbations of network activity [94] will be required to provide direct causal evidence for the low-dimensional attractor dynamics predicted by CAN models and should provide the final proof of the essential ingredients required for building grid cells in the mammalian MEC.
